# Association of preadmission metformin use and mortality in patients with sepsis and diabetes mellitus: a systematic review and meta-analysis of cohort studies

**DOI:** 10.1186/s13054-019-2346-4

**Published:** 2019-02-18

**Authors:** Huoyan Liang, Xianfei Ding, Lifeng Li, Tian Wang, Quancheng Kan, Lexin Wang, Tongwen Sun

**Affiliations:** 1 General ICU, The First Affiliated Hospital of Zhengzhou University, Henan Key Laboratory of Critical Care Medicine, Zhengzhou, 450052 China; 2 Biotherapy Center, The First Affiliated Hospital of Zhengzhou University, Zhengzhou, 450052 China; 3 Department of Pharmacy, The First Affiliated Hospital of Zhengzhou University, Zhengzhou, 450052 China; 4 School of Biomedical Sciences, Charles Sturt University, Wagga Wagga, NSW 2650 Australia

**Keywords:** Metformin, Mortality, Sepsis, Diabetes mellitus, Meta-analysis, Systematic review

## Abstract

**Background:**

Recent studies have reported that preadmission metformin users had lower mortality than non-metformin users in patients with sepsis and diabetes mellitus; however, these results are still controversial. Therefore, we conducted a systematic review and meta-analysis of published observational cohort data to determine the association between preadmission metformin use and mortality in septic adult patients with diabetes mellitus.

**Methods:**

The MEDLINE, EMBASE, and Cochrane CENTRAL databases were searched from their inception to September 30, 2018. Cohort studies that evaluated the use of metformin in septic adult patients with diabetes mellitus were included. The quality of outcomes was evaluated using the Newcastle-Ottawa Scale (NOS). The inverse variance method with random effects modelling was used to calculate the pooled odds ratios (ORs) and 95% CIs.

**Results:**

Five observational cohort studies (1282 patients) that were all judged as having a low risk of bias were included. In this meta-analysis, metformin use was associated with a significantly lower mortality rate (OR, 0.59; 95% CI, 0.43–0.79, *P* = 0.001).

**Conclusions:**

This meta-analysis indicated an association between metformin use prior to admission and lower mortality in septic adult patients with diabetes mellitus. This finding suggested that the possible effect of metformin should be evaluated in future clinical trials.

## Introduction

Sepsis is a life-threatening organ dysfunction caused by dysregulated host responses to infection [1]. Because the high mortality due to sepsis remains a major medical problem [2, 3], exploring the mechanism of its development is particularly important. To date, the exact mechanism remains unclear, but it is widely postulated that the release of inflammatory factors by innate immune cells plays an important role in sepsis disease pathogenesis [4, 5]. Interestingly, recent studies indicated that the active molecular responses in inflammation require intensive metabolic support, and modulation of the metabolic pathways could become a novel strategy to restrict inflammatory diseases [6].

Metformin is a reagent with extensive and strong metabolic regulatory activities, and it is often used as a first-line antidiabetic drug for the treatment of type 2 diabetes [7, 8]. In addition to its well-known hypoglycaemic activities, increasing evidence has suggested that metformin inhibits the expression of pro-inflammatory factors in vitro and ameliorates inflammatory injuries in vivo [9–14]. The mechanisms underlying the pharmacological effects of metformin remain unknown. It has been suggested that metformin inhibits the activity of the mitochondrial respiratory complex, which decreases the generation of ATP and activates adenosine 5′-monophosphate (AMP)-activated protein kinase (AMPK) [15]. Furthermore, several studies have suggested that AMPK activation by drugs or small molecular compounds protects against experimental sepsis in animals [16–18], demonstrating that AMPK plays an irreplaceable role in the pathogenesis of sepsis. Moreover, AMPK has been regarded as a major target mediating the effects of metformin.

Given the evidence of AMPK activation by metformin, metformin is a potential treatment for sepsis. In recent years, some studies [19, 20] have suggested that in patients with sepsis and diabetes mellitus, the preadmission metformin users had higher lactate levels but lower mortality than the non-metformin users. Geen et al. [21] reported similar lactate levels between preadmission metformin users and non-metformin users, but metformin users still had significantly lower mortality than nonusers. However, some studies [22, 23] showed that preadmission metformin use had no significant effect on mortality compared with non-metformin use in septic patients with diabetes mellitus. Therefore, it is necessary to consolidate the available information to assess whether metformin is beneficial for improving the outcomes of sepsis in patients with diabetes mellitus.

## Methods

### Search strategy

**Fig. 1:**
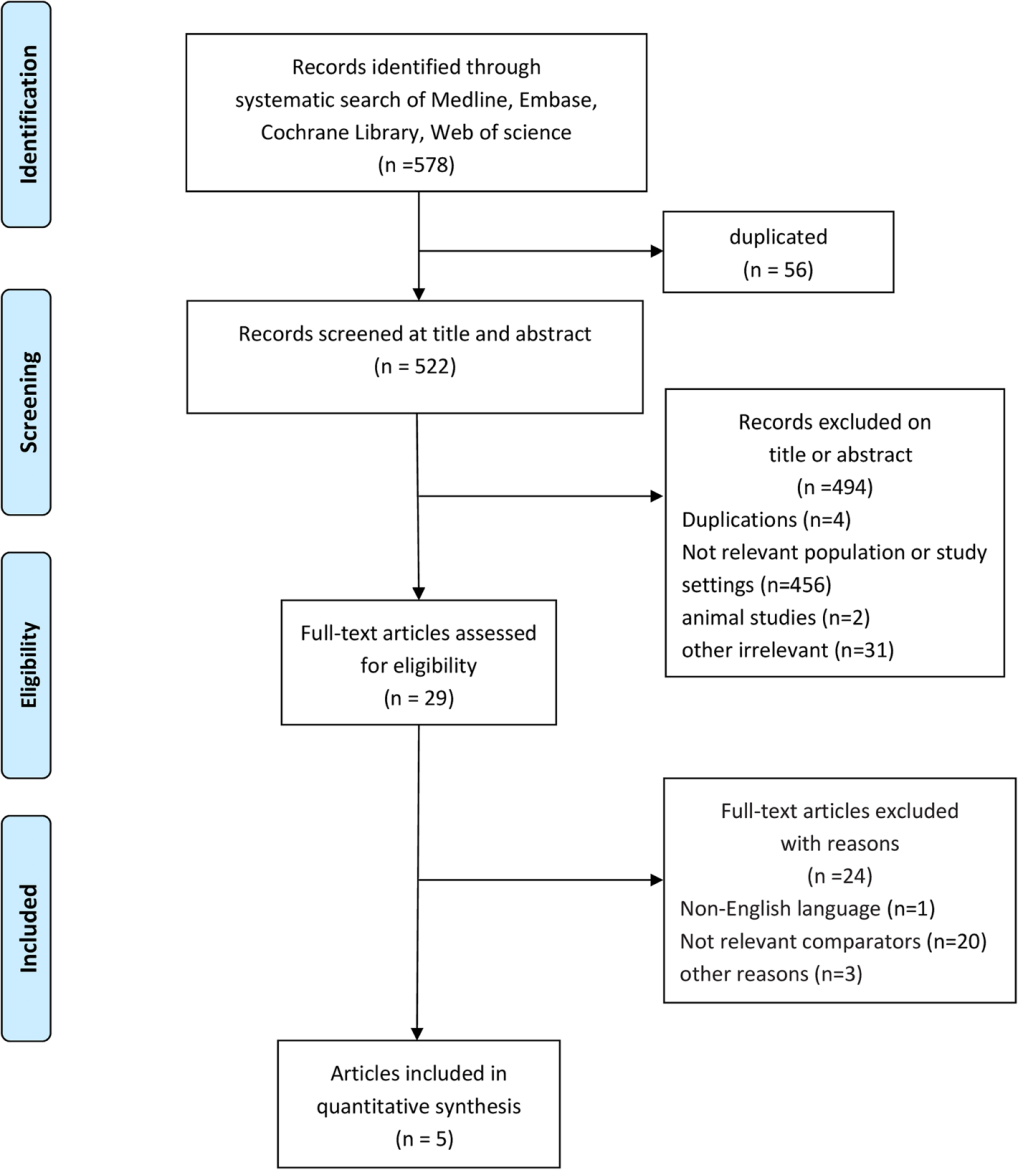
Flowchart of the study selection process

The methods complied with the meta-analysis of Observational Studies in Epidemiology guidelines [24], and the study profile is presented in Fig. 1. We searched the MEDLINE, EMBASE, and Cochrane CENTRAL databases for English language articles published from the inception of the database to September 30, 2018. A combination of MeSH/Emtree and title/abstract keywords was used. The search terms were “metformin”, “sepsis,” “severe sepsis”, “septic shock”, “mortality”, and “death”.

### Eligibility criteria

Studies were considered suitable for inclusion in this meta-analysis if (1) they enrolled septic patients with diabetes mellitus who used metformin, (2) the comparative arms were septic patients with or without diabetes mellitus, (3) they measured the mortality of metformin users and non-metformin users, (4) all the patients were adults, (5) they were observational studies from all settings (emergency department, hospital ward, or ICU), and (6) they were written in English. If the studies lacked outcome data or provided only the mortality of patients with sepsis complicated with other illnesses, they were excluded. If the full text could not be retrieved or if the article was a commentary or a review, it was excluded.

### Selection of studies and data extraction

All the available data were extracted from each study by two investigators independently according to the aforementioned inclusion criteria, and any differences were resolved by discussion with a third investigator. The following data were collected from each study: the name of the first author, publication year, country where the research was performed, study design, number of patients, sex, mean age, primary outcome, follow-up, and unadjusted and adjusted odds ratios (ORs) and 95% CIs of the primary outcome.

### Risk of bias assessment

The risk of bias was assessed for each outcome in all included studies using the Newcastle-Ottawa Scale (NOS) for cohort studies [25]. A maximum of nine points were awarded based on the cohort selection (maximum four points), the comparability of the cohort design and analysis (maximum two points), and the adequacy of the outcome measures (maximum three points); seven to nine points was considered high quality [25].

### Statistical analysis

The outcome of interest was the mortality of patients with sepsis with preadmission metformin use vs. non-metformin use. This meta-analysis used the pooled effect of each outcome. To investigate the heterogeneity between studies, we used a fixed effects model to calculate odds ratios (ORs) and 95% CIs for each outcome. Heterogeneity was assessed using *I*^*2*^ and *P* values, and the percentage of variability that was due to heterogeneity rather than sampling error (13, 14) was considered moderate when *I*^*2*^ equalled 51–74% and high when *I*^*2*^ was greater than or equal to 75%. Begg’s funnel plot [26] and Egger’s linear regression [27] were used to assess potential publication bias. Funnel plots were visually assessed for asymmetry. For Egger’s tests, *P* < 0.1 indicated a significantly small study size. All statistical analyses were performed using STATA 14.0 (College Station, Texas 77845, USA, Serial number: 401406267051).

## Results

### Study selection

We initially identified 578 records, and 56 articles remained after duplicates were removed. After preliminary screening by title and abstract, 29 cohort studies were identified that appeared to address issues potentially related to the primary study question. However, only 5 articles [19–23] enrolling 1282 patients were ultimately included in this meta-analysis after the study selection process (Fig. 1).

### Study characteristics

All included articles were observational cohort studies that reported patients with sepsis and diabetes mellitus who used metformin preadmission [19–23]. Three studies [19, 20, 23] reported in-hospital mortality as the primary outcome, and the remaining two studies [21, 22] reported 28-day mortality as the primary outcome. Then, we extracted the unadjusted and adjusted ORs and 95% CIs of the primary outcome data. If missing, the data were calculated based on the raw data provided in the study. Baseline information about the analysed studies is presented in Table 1**.**

**Table 1:** Summary of identified studies

First author	Year	Country	Study design	Multi/single centre	Number of metformin use in patients	Number of non-metformin use in patients	Female/male of metformin use in patients	Female/male of non-metformin use in patients	Mean age of metformin use in patients	Mean age of non-metformin use in patients	Study period	Primary Outcome	Unadjusted OR (95% CI)	Adjusted OR (95% CI)
Doenyas-Bara [19]	2016	Israel	RC	Single	44	118	22/22	47/71	67	68	01/2011–06/2013	In-hospital mortality	0.42(0.27–0.66)	0.21 (0.05–0.94)
Green [21]	2012	America	RC	Single	192	343	NA	NA	71	72	02/2007–10/2008	28-day mortality	NA	0.38 (0.19–0.76)
Jochmans [20]	2017	French	RC	Single	52	79	19/33	23/56	74	68.4	10/2010–12/2013	In-hospital mortality	0.87 (0.43–1.78)	0.61 (0.36–0.99)
Park [22]	2017	Korea	RC	Single	71	142	32/39	62/80	71	66	08/2008–09/2014	28-day mortality	0.71 (0.32–1.58)	1.09 (0.41–2.85)
van Vught [23]	2016	Holland	OC	Multi	114	127	36/78	61/66	67	65.1	01/2011–07/2013	In-hospital mortality	0.69 (0.40–1.19)	NA

Abbreviations: *RC* retrospective cohort, *OC* observational cohort, *OR* odds ratio, *CI* confidence interval, *NA* not reported

### Risk of bias assessment

The five eligible studies were retrospective cohort studies, all of which had greater than or equal to seven points and showed a low risk of bias according to the NOS. Specific details of the risk of bias in the included studies are reported in Table 2**.**

**Table 2:** The Newcastle-Ottawa quality assessment scale of including studies

Studies	Selection			Comparability			Assessment of outcome			Total quality score
First author	Representativeness of exposure arm(s)	Selection of the comparative arm(s)	Origin of exposure source	Demonstration that outcome of interest was not present at start of study	Studies controlling the most important factors	Studies controlling the other main factors	Assessment of outcome with independency	Adequacy of follow-up length (to assess outcome)	Lost to follow-up acceptable (less than 10% and reported)	
Doenyas-Bara [19]	*	*	*	*	*	*	*	*		8
Green [21]	*	*	*	*	*	*	*	*		8
Jochmans [20]	*	*	*	*	*	*	*	*		8
Park [22]	*	*	*	*	*	*	*	*		8
van Vught [23]	*	*	*	*	*	–	*	*		7

### Effects of metformin on outcomes

**Fig. 2:**
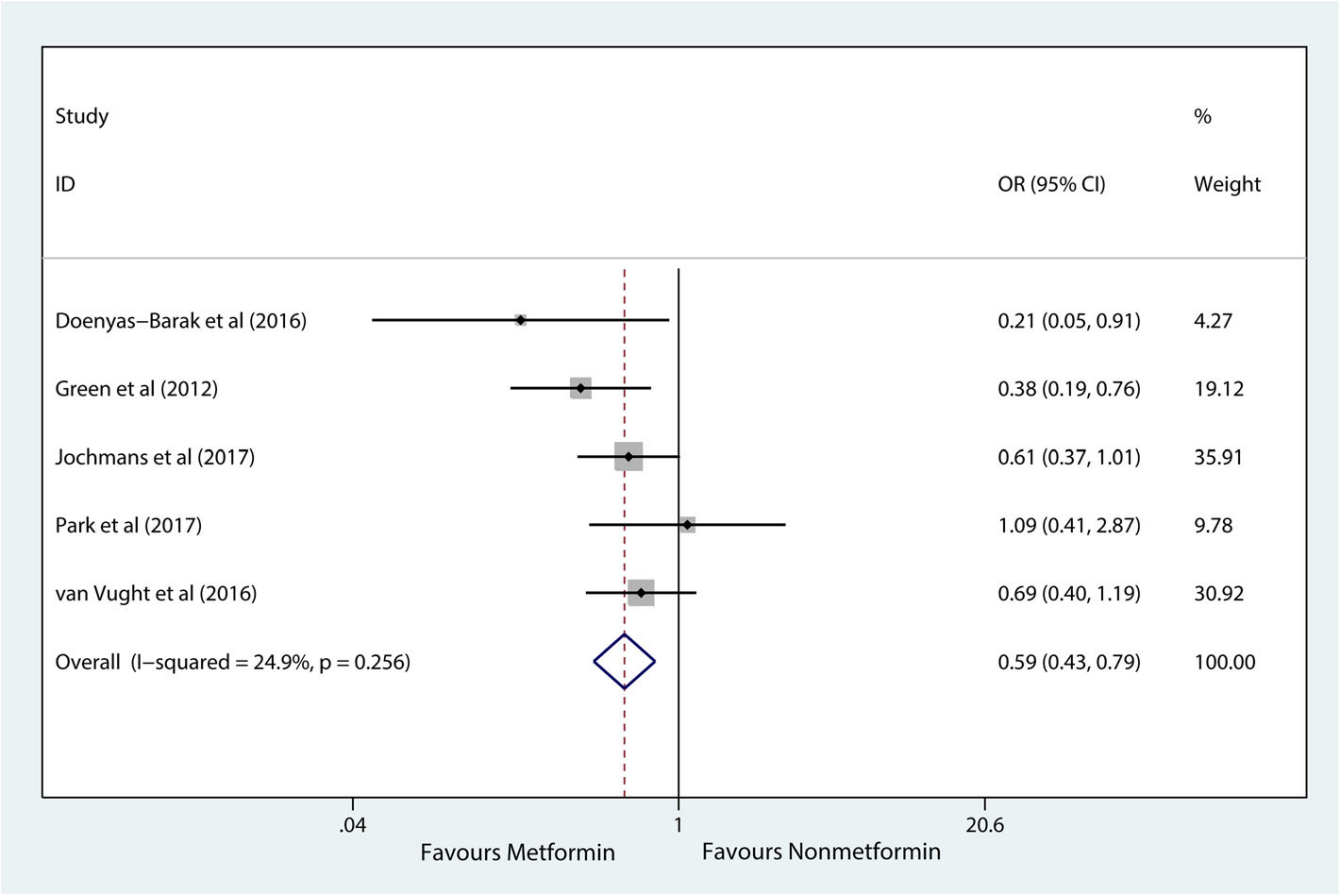
Meta-analysis of the overall pooled odds ratios (ORs) of studies investigating the mortality outcomes of patients with sepsis with diabetes mellitus. Forest plot showing the significance of the association between metformin use and mortality in septic patients with diabetes according to the fixed effects model

In the five included cohort studies (1282 patients), preadmission metformin users had significantly lower mortality than nonusers among patients with sepsis (OR, 0.59; 95% CI, 0.43–0.79, *P* = 0.001) (Fig. 2) [19–23]. Because few studies have investigated the association of metformin with the length of hospital stay and other outcomes, a meta-analysis of secondary outcomes was not conducted.

### Sensitivity analyses

**Fig. 3:**
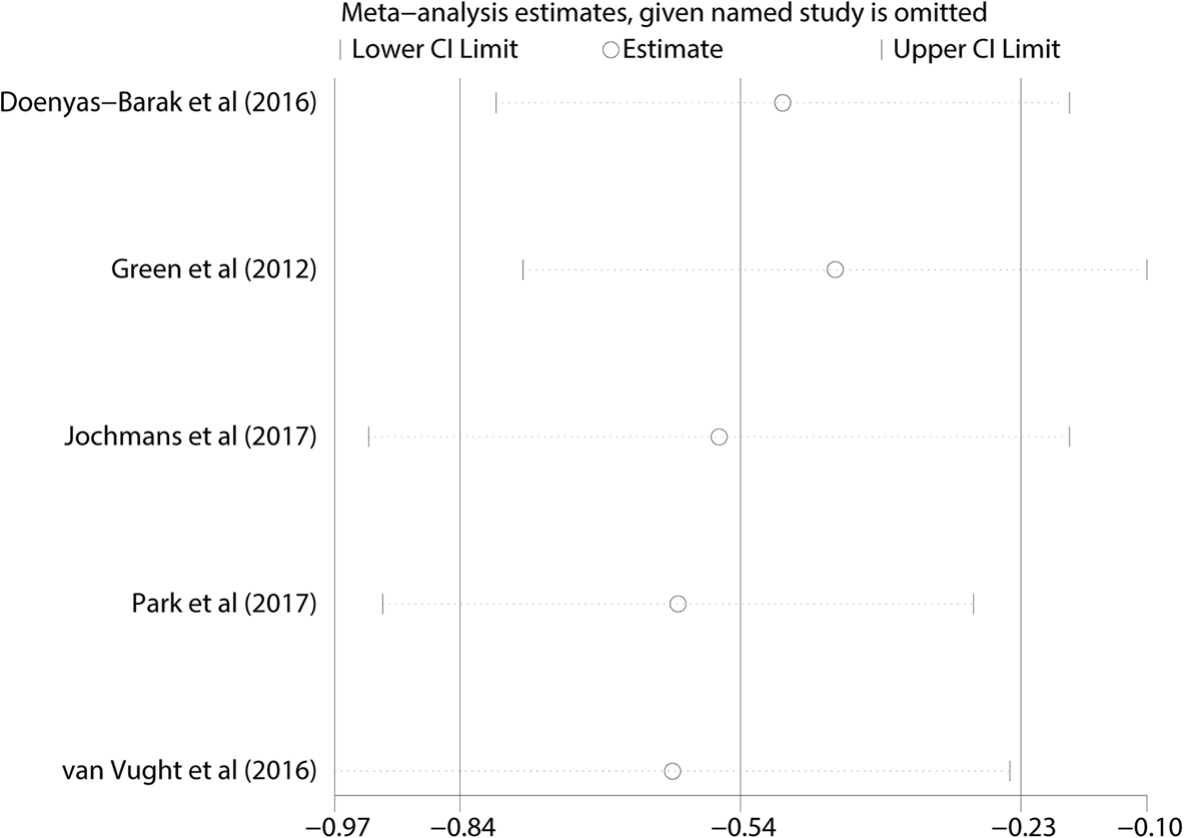
The sensitivity analysis showed that the studies were robust and reliable regarding the use of metformin and the mortality of patients with sepsis with diabetes mellitus. The analysis was performed by excluding one study at a time and calculating the pooled estimate for the remaining studies

Because all included studies were observational cohort studies with a low risk of bias **(**Table 1**)**, a sensitivity analysis based on the methodological criteria was not conducted. A sensitivity analysis was performed only to assess the influence of any one study on the pooled OR and 95% CI by omitting one individual study at a time. Our findings showed that the results were robust and reliable (Fig. 3). In addition, based on the analysis of metformin users stratified by age, the heterogeneity of the overall estimate in each subgroup was not significant.

### Evaluation of publication bias

**Fig. 4:**
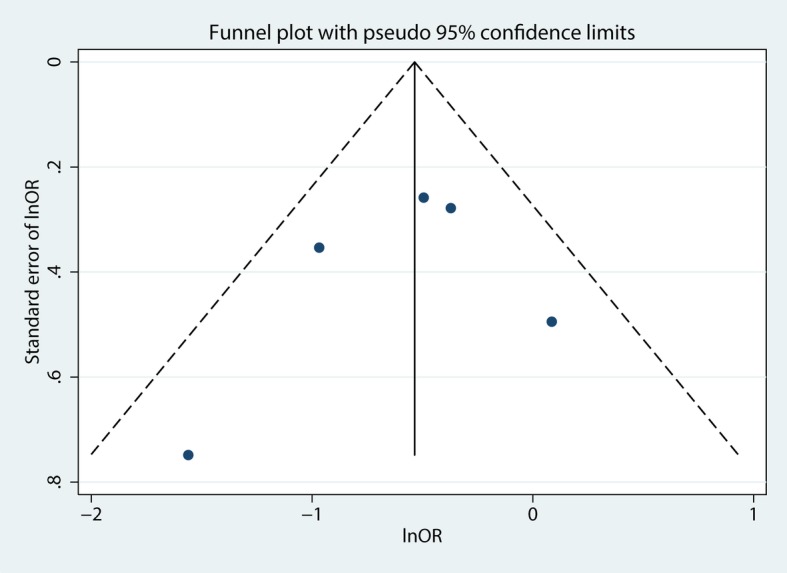
Funnel plot evaluating mortality after the preadmission use of metformin in septic patients with diabetes mellitus

Funnel plots (Fig. 4) and Egger’s regression asymmetry tests were performed to evaluate the publication bias in these five studies evaluating mortality after metformin use in septic patients with diabetes mellitus. No significant publication bias was found (*P* = 0.570).

## Discussion

This meta-analysis included 1282 patients and demonstrated that preadmission metformin users had lower mortality than nonusers in patients with sepsis. This is the first systematic review and meta-analysis to describe the association between preadmission metformin use and mortality in septic adult patients with diabetes mellitus. Our finding indicates that metformin may have therapeutic potential for patients with sepsis and diabetes mellitus.

A beneficial association between metformin and mortality has already been described in selected ICU patients with chronic heart failure and liver disease [28–31], and a retrospective study [31] of 17 Danish ICUs found that 30-day mortality was lower in metformin users than in non-metformin users, which was the same result as that found in this meta-analysis. In addition, many experimental studies [32–37] have reported that metformin can ameliorate sepsis or endotoxaemia-induced injuries in various organs and many inflammatory diseases. For example, Wu et al. [38] reported that metformin can alleviate acute lung injury, and Liu et al. [13] showed that metformin can attenuate acute myocarditis. However, some studies [39, 40] have shown that metformin cannot decrease but instead increases the mortality rate of septic animals. And unfortunately, to date, the association between preadmission metformin use and mortality in septic patients with diabetes mellitus remains controversial. Some studies [20, 22, 23] have shown that in patients with sepsis, preadmission metformin users were not significantly different from nonusers, whereas the studies by Green et al. [21] and Doenyas-Bara et al. [19] suggested that the mortality was significantly lower in preadmission metformin users than in nonusers among patients with sepsis. Therefore, this meta-analysis provides evidence to support that preadmission metformin use may decrease mortality in septic patients with diabetes mellitus.

The mechanism underlying the ability of metformin to decrease the mortality of septic patients remains unclear. Metformin may supply higher amounts of lactate, serving as an energetic carbon source, thus making energy available to ischaemic tissue; this hypothesis is consistent with the results of a study [41] that indicated that lactic acid is also a key energy source, like glucose, amino acids, and ketones. Furthermore, Friesecke et al. [42] and Protti et al. [43] suggested that lactic acidosis due to metformin use is associated with lower mortality compared to other forms of lactic acidosis, which may explain why preadmission metformin users had lower mortality than nonusers among patients with sepsis and diabetes mellitus. Moreover, the mechanism may also be related to the potential protective effects of metformin, including its anti-endotoxaemic, anti-inflammatory, and vasoactive properties [44–46]. Additionally, metformin has been shown to decrease the expression of nitric oxide synthase in animal models, which could ameliorate vasodilatation [47]. Most importantly, metformin induces the activation of AMPK, an enzyme that is a key cellular energy sensor that maintains energy homeostasis at the cellular and organismal levels [48]. Once activated, AMPK regulates cellular energy status, switching on the ATP-generating pathways and switching off the ATP-consuming pathways [49]. Furthermore, some studies [50, 51] have shown that metformin may possess antimicrobial effects, which may be the primary mechanism involved in the beneficial effects on sepsis. As a result, cellular function is improved under stressful conditions, leading to improved cardiovascular function and hypoxia. These potential protective effects of metformin use may significantly ameliorate the prognosis of septic patients, but this association requires further study.

Meta-analysis is a method used for the comprehensive statistical analysis of multiple studies on a topic. When the difference between the outcomes of each study is greater than expected, statistical heterogeneity is present in the pooled results of the meta-analysis. The four included cohort studies had a high degree of heterogeneity, reflecting differences in these studies. In this meta-analysis, the risk of bias assessment of the included studies showed a low risk bias. Therefore, the statistical heterogeneity was not considered methodological heterogeneity. The heterogeneity may be derived from many causes, such as the small sample sizes, the different initial lactate levels and the use of other antidiabetic medications.

This meta-analysis has several strengths. In the study by Doenyas-Bara et al. [19], the metformin users had more severe diabetes mellitus at admission than the non-metformin users, thus providing additional strong evidence to support the outcome that metformin users may have lower mortality among patients with sepsis and diabetes mellitus. Second, the risk of bias assessment used the NOS, which showed a low risk of bias among the included studies. Third, we used the random effects model with generic inverse variance, and we extracted the adjusted ORs and 95% CIs so that the pooled estimate of the OR for the effect of metformin on mortality could be computed. Finally, the outcome of the sensitivity analysis showed that this result was robust and reliable.

However, this study has important limitations. Although we did our best to conduct a comprehensive search of the relevant literature, only four studies met our inclusion criteria, so the conclusion may need more original studies for confirmation. Second, the sample size of the septic patients with diabetes mellitus was small, suggesting that the result may not be reliable. Nevertheless, the robustness of our conclusions was supported by the result of the risk of bias assessment. Third, the outcome of this meta-analysis may be confounded by the fact that the inclusion criteria of the four studies varied considerably. Fourth, we studied the relationship between metformin use prior to admission and mortality in septic patients with diabetes mellitus, so it is not clear whether metformin is beneficial during the hospital stay for septic patients with diabetes mellitus previously untreated with metformin; therefore, further studies are needed. Fifth, although septic patients with diabetes may benefit from preadmission metformin use, the effects and safety of metformin treatment initiation and continuation in patients who are critically ill remain to be further clarified. Finally, there were no randomised controlled trials included in this meta-analysis, which is a limitation even though all the included retrospective cohort studies had a low risk of bias.

## Conclusions

This is the first systematic review and meta-analysis to describe the association of preadmission metformin use and mortality in septic adult patients with diabetes mellitus. The results suggest that in septic adult patients with diabetes mellitus, preadmission metformin use is associated with lower mortality. However, the current conclusions need to be further supported by more high-quality original studies.

## Abbreviations

AMPK: Adenosine 5′-monophosphate (AMP)-activated protein kinase
CI: Confidence interval
NOS: Newcastle-Ottawa Scale
OR: Odds ratio
RC: Retrospective cohort
